# Psoriatic Arthritis: An Update

**DOI:** 10.1155/2012/176298

**Published:** 2012-10-17

**Authors:** Peter Lloyd, Caitriona Ryan, Alan Menter

**Affiliations:** ^1^Department of Internal Medicine, University of Chicago (NorthShore), 2650 Ridge Avenue, Evanston, IL 60201, USA; ^2^Department of Dermatology, Baylor University Medical Center, Dallas, TX 75246, USA

## Abstract

Psoriatic arthritis is a debilitating condition, which affects approximately one-quarter of psoriasis patients. Recent findings have furthered our understanding of the complex pathophysiology of PsA. There have been major advances in the identification of genes associated with joint involvement but not with cutaneous disease alone. The elucidation of key immunologic pathways has allowed the development of novel targeted therapies that are in the research pipeline. Currently, good screening tests and biomarkers to diagnose early PsA and to guide therapy are limited. In this paper, we present recent findings with regard to the immunopathogenesis and genetics of PsA, biomarkers, and screening tools and review the targeted therapies currently in clinical trials.

## 1. Introduction

Psoriatic arthritis (PsA) is an inflammatory arthropathy, which is associated with psoriasis in approximately 25% of patients. It is characterized by stiffness, pain, swelling, and tenderness of the joints as well as the surrounding ligaments and tendons [1, 2]. It affects men and women equally and typically presents at the age of 30 to 50 years [2]. Cutaneous disease usually precedes the onset of PsA by an average of 10 years in the majority of patients but 14–21% of patients with PsA develop symptoms of arthritis prior to the development of skin disease [2]. Psoriatic arthritis is classified as a seronegative spondyloarthritis due to the potential axial involvement, the contribution of enthesitis to its pathogenesis, and increased association with HLA-B27 [3]. The presentation is variable and can range from a mild, nondestructive arthritis to a severe, debilitating, erosive arthropathy. 

There are multiple clinical subsets as defined by Moll and Wright: monoarthritis of the large joints, distal interphalangeal arthritis, spondyloarthritis, or a symmetrical deforming polyarthropathy much akin to that of rheumatoid arthritis. Left untreated, a proportion of patients may develop persistent inflammation with deforming progressive joint damage which leads to severe physical limitation and disability [3]. In many patients articular patterns change or overlap in time [2]. Enthesitis may occur at any site, but more commonly at the insertion sites of the plantar fascia, the Achilles tendons, and ligamentous attachments to the ribs, spine, and pelvis [4]. Dactylitis, an important feature of PsA, is a combination of enthesitis of the tendons and ligaments and synovitis involving all joints in the digit. The severity of the skin and joint disease frequently do not correlate with each other. Although in the past it was always thought that the presence of nail psoriasis correlated with the development of psoriatic arthritis, more recent evidence does not support this [5]. Ocular manifestations of PsA include conjunctivitis, iritis, and urethritis. Radiographic characteristics of PsA include the development of erosions, the presence of pencil-in-cup deformity, arthritis mutilans, spur formation, nonmarginal asymmetric syndesmophytes, and asymmetric sacroiliitis. 

In the past decade, considerable progress has been made in further elucidating the immunologic and genetic basis of PsA, and defining its clinical and epidemiological characteristics. More importantly, there have been significant advances in the development of more targeted systemic and biologic treatments for PsA. This update review advances in the field of psoriatic arthritis in the past decade and discusses the future direction of PsA research and therapy. 

## 2. Advances in Epidemiology

It is imperative to diagnose psoriatic arthritis at its first onset because early diagnosis and treatment may reduce irreversible joint damage. Patients with PsA who were started on etanercept within two years of disease onset had a more significant improvement in pain assessments than those who had PsA for more than two years prior to commencing etanercept [6]. Additionally, the SwePsA registry found that the early diagnosis of psoriatic arthritis was associated with lower joint disease activity at the 5-year follow-up time point [7]. In this study, male gender, axial disease, preserved function at diagnosis and lower baseline health assessment questionnaire scores also portended a better prognosis. Surprisingly, it was also shown that male gender was a predictor of more rapid radiological progression despite a better clinical outcome [8].

## 3. Advances in Screening and Biomarkers of Disease Activity

Early detection of PsA is difficult in the absence of a validated screening test or biomarkers of disease activity. The Classification of Psoriatic Arthritis (CASPAR) system is the most widely used criteria for diagnosis. Unfortunately, there are many patients with undiagnosed PsA [2]. For example, in one study, 29% of the psoriatic patients in dermatology clinics in Dublin had undiagnosed PsA, while in another study more than one-third of patients seen in 34 dermatology centers in Europe and North America had undiagnosed PsA [9, 10]. As a result, screening for and early detection of psoriatic arthritis must improve. 

Korotaeva et al. showed that early detection of PsA can be accomplished by the combination of a clinical exam and magnetic resonance imaging (MRI), and that MRI may be superior in diagnosing tenosynovitis [11]. One study showed that patients with active PsA have elevated total serum IgG and more cell surface bound IgG, making Fc receptors possible biomarkers of disease activity [12]. Interleukin-23 (IL-23) serum concentration has also been evaluated as a possible biomarker of disease activity, but no correlation with disease activity was found [13]. Another study compared the serum levels of various potential biomarkers and the presence of scalp and nail involvement, in patients with cutaneous disease alone and patients with joint involvement [14]. It was shown that a tool incorporating high sensitivity C reactive protein, osteoprotegerin (OPG), matrix metalloproteinase 3 (MMP-3), and the ratio of C-propeptide of Type II collagen (CPII) to collagen fragment neoepitopes Col2-3/4(long mono) (C2C), in association with the presence of nail and scalp psoriasis could be helpful in screening for PsA [14]. Further studies to determine the best screening method are warranted, as early diagnosis significantly decreases morbidity and increases the quality of life in patients with PsA.

Currently, there is no validated disease activity score for psoriatic arthritis. The two most promising measures are the modified composite psoriatic disease activity index (mCPDAI) and the arithmetic mean desirability function (AMDF). The mCPDAI is a composite score of 4 domains: joints, skin, dactylitis, and enthesitis. The AMDF is calculated from the cutaneous involvement, joint, and global VAS assessment. Both of these appear to correlate best with disease activity [9].

## 4. Comorbidities

It is now well known that psoriatic patients are at higher risk for the metabolic syndrome and thus have a larger waist circumference. It was recently shown, however, that there is no correlation between a larger waist circumference and more severe PsA [15, 16]. Interestingly, a prospective study of 135 obese and 135 nonobese PsA patients starting tumor necrosis factor-alpha (TNF-*α*) inhibitors showed that obese patients were less likely to achieve minimal disease activity (MDA) (hazard ratio: 4.90, 95% CI : 3.04–7.87, *P* < 0.001). Of those who achieved MDA, obese patients were more likely to relapse at 24 months [17]. 

 Another prospective study among 138 obese PsA patients commencing TNF-*α* inhibitors on a self-managed diet or hypocaloric diet showed that the adherence to a hypocaloric, fiber-enriched diet associated greater likelihood of achieving MDA [18]. Studies have shown that patients with psoriasis, gout, and rheumatoid arthritis have an increased risk of acute myocardial infarction [19]. Using the Kaiser Permanente healthcare database in California, a retrospective cohort study of 24,081 psoriasis and PsA patients examined the effect of TNF-*α* inhibitors on the incidence of myocardial infarction [20]. A multivariant analysis was performed to control for cardiovascular risk factors. Patients receiving TNF-*α* inhibitors had a 48% reduction in the risk of myocardial infarction (*P* = 0.0062) [20]. The presence joint involvement increased the risk of myocardial infarction by 42% [20]. 

## 5. Advances in Understanding the Immunologic Pathogenesis of Psoriatic Arthritis

Current literature suggests that both acquired and innate immunity are responsible for the development of PsA [21, 22]. Until recently, psoriasis and PsA were thought to be predominately Th1-cell mediated diseases based on the large number of interferon-gamma producing cells found in cutaneous eruptions [23]. Recent studies have proposed that the T helper 17 (TH17) cell plays a more pivotal role in the pathology of psoriasis and PsA. T helper 17 cells are a subset of helper T cells and differ functionally from Th1 and T helper 2 (TH2) cells [23]. TH17 cells induce acquired immune responses against microbes and produce interleukin (IL) 17A, IL-17F, IL-21, and IL-22 [24].

Tumor necrosis factor-alpha, interferon gamma, interferon-alpha, IL-6, and IL-1beta induce the secretion of IL-12 and IL-23 by myeloid dendritic cells which cause the differentiation of TH-1 and TH17 cells, respectively, [25]. TH17 cells then produce IL-17 which induces the production of proinflammatory cytokines and angiogenic factors [25]. It also commits naive T cells to the TH17 lineage thus creating a positive feedback loop for Th17 inflammation [25]. Interleukin-17 and IL-12 along with the TH1 cytokines (TNF-*α*, IL-1B, and IL-10) have been found in higher levels in psoriatic arthritis synovium and IL-17 and IL-23 have been implicated in the potentiation of osteoclastogenesis and bone erosion in psoriatic arthritis joints [26–28].

A key intracellular regulator of the innate immune system is nuclear factor kappa beta (NF*κ*B) which is activated by TNF-*α*, IL-1, and IL-17 [29]. Once activated, NF*κ*B triggers the transcription of several genes involved in the pathogenesis of inflammatory diseases such as PsA [22]. Tumor necrosis factor-alpha also induces the expression of receptor activator of nuclear factor *κ*B (RANK) which is required for the receptor activator of nuclear factor *κ*B ligand (RANKL) to trigger the differentiation of osteoclast precursor cells into activated osteoclasts [22]. In psoriatic synovial tissues, RANKL is markedly upregulated and its natural antagonist osteoprotegerin is downregulated [30]. RANKL is also expressed in the fibroblastoid cells of the synovial lining and by the T cells infiltrating the synovium, providing an additional stimulus to activate osteoclasts [30]. This upregulation of activated osteoclasts causes bone resorption which leads to the manifestations of psoriatic arthritis [30]. 

## 6. Genetics

 It was once thought that psoriasis and psoriatic arthritis were a continuum resulting from key genetic polymorphism. Recent studies have shown that there are distinct genetic differences between the two diseases. Population-based studies have shown that the heritability of PsA is 3–5 times higher than that of psoriasis [22, 31]. Overall, the gene MICA*002 is more specific to PsA than psoriasis and certain genes have been found to be more frequently associated with specific types of PsA [32]. For example, HLA-B38 and HLA-B39 are more frequent in peripheral PsA and HLA-B27 is more frequently seen in PsA with spondylitis [32]. Psoriatic arthritis patients with HLA-B27 or DQB1*02 were shown to have an increased risk of developing arthritis mutilans, the most severe form of psoriatic arthritis [33]. Additionally, HLA-B*39, HLA-B*27, and HLA-A*02 and the KIR gene *KIR3DS1 *were found to be independently associated with the increased progression of peripheral joint damage, whereas the alleles DQB1*0604, C*04, and B*50 were associated with decreases progression [34].

The IL-13 locus associates with PsA, but not psoriasis [22]. Bowes et al. found two IL-13 single nucleotide polymorphisms (SNPs), rs1800925, and rs20541 with the major allele, rs1800925, conferring disease susceptibility [35]. This association has been reported previously but contradicts the findings of Nair et al. who showed that IL-13 has a significant association with both psoriasis and PsA [36]. Another SNP, rs104 8455, at the class I region of MHC just 34.7 kb upstream from HLA-C has also been linked to psoriatic arthritis [37]. Additionally, genomewide association study (GWAS) data has shown that the rs10782001 variant of FBXL19, a gene in the NF*κ*B network, is more frequent in PsA and the effect size of the TRAF3 interacting protein (TRAF31P2) in the Th17 pathway, to be mildly higher in PsA [38, 39]. The GWAS has also identified variants in several genes in the NF*κ*B signaling network, including TNFAIP3, TNIP1, NFKBIA, REL, and NOS2, which have been implicated in PsA [40–43]. VEGF gene polymorphisms have also been found to be associated with different psoriatic arthritis phenotypes. For example, the peripheral joint involvement of psoriatic arthritis was associated with the VEGF polymorphism C(-2578)A [44].

## 7. Therapy

Nonsteroidal anti-inflammatory drugs (NSAIDs) help with symptomatic relief, but they do not alter the disease course or prevent disease progression. Intra-articular steroid injections can be used for symptomatic relief. In psoriatic arthritis, dramatic flares in skin disease have been reported with corticosteroid taper; therefore, systemic corticosteroids ideally should be avoided in this patient population [3]. Physical therapy may also be helpful in symptomatic relief. Disease modifying anti-rheumatic drugs (DMARDs) are the main-stay of treatment for patients suffering from PsA. Traditional oral agents include methotrexate, sulfasalazine, cyclosporine and leflunomide. The TNF-*α* inhibitors include etanercept, infliximab, adalimumab, and golimumab. Currently, the most effective class of therapeutic agents for treating PsA is the TNF-*α* inhibitors; however, these drugs show a 30 to 40% primary failure rate in both randomized clinical trials and registry-based longitudinal studies [45–47].

A recent phase III trial of ustekinumab, an IL-12/23 inhibitor, for PsA showed ACR-20 responses of 42.4% for ustekinumab 45 mg weekly and 49.5% for ustekinumab 90 mg weekly compared to 22.8% for placebo at 24 weeks [48]. Even though an ACR-20 of nearly 50% was obtained with 90 mg of ustekinumab weekly; etanercept produces an ACR20 of 59% at 24 weeks, adalimumab an ACR20 of 58% at 12 weeks which is maintained to 24 weeks, and infliximab an ACR20 of 58% at week 14 which was maintained to 24 weeks [49–51].

Secukinumab (AIN457, Novartis) a fully human anti-interleukin-17A antibody was evaluated in 18 patients with psoriatic arthritis. Although there was some improvement in clinical scores, the primary endpoint of the proportion of ACR20 responders at week 6 was not met with 39% and 23% of patients treated with secukinumab versus placebo achieving an ACR-20 response (*P* = 0.27) [52]. The safety profile of secukinumab was favorable and the most common adverse events were headache and gastrointestinal disturbance [52]. It is currently undergoing a phase III trial which is evaluating the efficacy and safety in PsA whom have not responded to current therapies. (http://www.clinicaltrials.gov/, NCT01169844). There is also a trial underway evaluating its long-term safety and efficacy up to two years in patients with active PsA. (http://www.clinicaltrials.gov/, NCT01392326).

Apremilast (CC-10004, Celgene) is an oral small molecule inhibitor of phosphodiesterase type 4 (PDE4), which results in decreased production of IL-2, IL-12, IFN gamma, TNF-*α*, leukotrienes, and decreased activity of nitric oxide synthase. A phase II, multicenter, randomized, double-blind, placebo controlled study included 204 subjects [53]. At week 12, 43.5% of subjects receiving apremilast 20 mg BID (*P* < 0,001) and 35.8% receiving 40 mg daily (*P* = 0.002) achieved ACR20 versus 11.8% receiving placebo [53]. The most common adverse events noted were gastrointestinal disturbance, upper respiratory tract infection, and headache [53]. The phase III, multicenter, randomized, double-blind, placebo controlled, parallel group, efficacy and safety study of two doses of apremilast in subjects with active PsA and a qualifying psoriasis lesion (PALACE-3) is currently underway. (http://www.clinicaltrials.gov/, NCT01212270). The primary outcome measure is the ACR20 and the results are to be released in the third quarter of 2012. 

Brodalumab (AMG827, Amgen) is a human monoclonal antibody, which binds to and blocks the signaling of the IL-17 receptor. The phase II trial is underway evaluating the safety and efficacy with the primary endpoint being the ACR20 at week 12. (http://www.clinicaltrials.gov/, NCT01516957). The primary completion date is December 2012.

RG4934 (Hoffmann-La Roche) is a specific anti-IL-17 monoclonal antibody in phase I studies for PsA with recruitment of the 19 patients completed in late 2011 [54].

## 8. Conclusions

Psoriatic arthritis is an inflammatory arthritis with a number of clinical patterns. Currently there are no validated screening serological methods to aid in early clinical diagnosis. Psoriatic arthritis is associated with different degrees of disability and an increased mortality risk especially when there is a delay in diagnosis. There are distinct genetic differences between psoriatic patients who develop PsA and those who remain free of joint involvement. This likely produces differences in the microenvironment of the skin and synovial tissues where inflammatory mediators are not identical. Treatment options include symptomatic as well as disease modifying agents either singly or in combination. Therapeutic agents beneficial for the cutaneous manifestations of psoriasis may not necessarily be equally efficacious for PsA and vice versa. However, even with the most effective agents there are still a significant percentage of treatment failures, creating the need for the further development of more effective and safer treatment options. Several medications are currently undergoing clinical trials and are showing promising results. Patients with PsA should be diagnosed early and treated promptly and aggressively in order to prevent joint destruction and poor clinical outcomes.
